# Diseases of the Nervous System

**Published:** 1893-08

**Authors:** 


### Book Notices.

---

DISEASES OF THE NERVOUS SYSTEM. A Text-book for Physicians and Students. By Ludwig Hirt, M. D., Professor at the University at Breslau. Translated, with permission of the author, by August Hoch, M. D., assisted by Frank R. Smith, A. M. (Cantab.), with an introduction by William Osler, M. D., F. R. C. P. pp. 683, with 178 illustrations. New York: D. Appleton & Co. 1893.

This work, known for several years in its original tongue, and received with favor by the profession as a disquisition upon diseases of the nervous system, comes to us in an excellent translation, as a competitor for appreciation as a text-book. Several features are notable, aside from the body of the text. The author prefaces each subject with a brief resume of the anatomical relations and the physiology of the portion of the nervous system discussed, assuming, wisely, it must be said, that such reviews will not be unacceptable to the majority of his readers. This feature might, in fact, be somewhat more elaborate without marring either the excellence of the work or the appearance of its pages; and the illustrations upon the anatoym of the parts described might with reason be made more comprehensive and comprehensible, if the work is to be thought of as a text-book for students. The arrangement of the subject matter is more or less novel. The author divides the book into three sections. In the first he discusses the diseases of the membranes of the brain, of the cranial nerves and of the brain proper. In the second section he takes up, in a similar manner, the diseases of the membranes of the cord, of the spinal nerves and of the cord proper. In the third section are discussed diseases of the general nervous system, first, functional, and second, organic. In the first of the latter divisions are grouped those functional nervous affections which are more or less localized in their implication of the system, as chorea, tetany, and paralysis agitans; in the second such as are more wide-spread in involvement of the body, as epilepsy, hysteria, and similar maladies. The organic diseases of the general nervous system are treated, multiple sclerosis, tabes dorsalis, paralytic dementia, and syphilis. In this arrangement there is, in every case, some excellent argument for deviation from the usual order; but one is forced to question whether it is a proper thing to deal in a text-book for stndents with locomotor ataxia as a disease of the general nervous system, and lose the value of emphasis of its spinal involvement, which is gained by the older classification. Exophthalmic goitre is placed where it probably belongs, among the diseases of the cranial (vagus) nerves; tetany, which has been produced as the result of infection, as, too, chorea, is placed among the functional diseases with more or less localized involvement of the general system.

The author, as is the case with most of his countrymen, is mindful of the minutiæ, sometimes to a degree which for a student might become embarrassing when compared with the slight attempts at emphasis of some of the more important points. Moreover, there are some few inaccuracies to be charged to the author.

One thing for which, perhaps, above all, the writer is to be applauded, is his position as to treatment. He states freely, where it is necessary, that little may be expected from treatment by medication, and he does not lead his reader a long chase through the pharmacopœa for remedies which are of no real value. One cannot but be charmed by the frankness and appreciation shown the intelligence of his readers. A. J. S.

